# Design and construction of a chimeric peptide, MeICT/IMe-AGAP, from two anti-cancer toxins of Iranian *Mesobuthus eupeus *scorpion 

**DOI:** 10.22099/mbrc.2023.46450.1804

**Published:** 2023

**Authors:** Razieh Seifi, Hoda Ayat, Ali Mohammad Ahadi

**Affiliations:** Department of Genetics, Shahrekord University, Rahbar Blvd, P.O Box 115 Shahrekord, Iran

**Keywords:** IMe-AGAP, MeICT, Anti-cancer peptides, Chimeric peptide, Scorpion toxins

## Abstract

Scorpion venom contains various toxin peptides with pharmacological and biological properties. Scorpion toxins specifically interact with membrane ion channels which play key roles in progression of cancer. Therefore, scorpion toxins have received special attention for targeting cancer cells. Two new toxins MeICT and IMe-AGAP, isolated from Iranian yellow scorpion, *Mesobuthus eupeus,* interact specifically with chloride and sodium channels, respectively. Anti-cancer properties of MeICT and IMe-AGAP have been determined before, in addition they show 81 and 93% similarity with two well-known anti-cancer toxins, CTX and AGAP, respectively. The aim of this study was construction of a fusion peptide MeICT/IMe-AGAP to target different ion channels involved in cancer progression. Design and structure of the fusion peptide were investigated by bioinformatics studies. Two fragments encoding MeICT and IMe-AGAP were fused using overlapping primers by SOEing-PCR. MeICT/IMe-AGAP chimeric fragment was cloned into pET32Rh vector, expressed in *Escherichia coli* host and analyzed by SDS-PAGE. The *in silico* studies showed that chimeric peptide with GPSPG linker preserved the three-dimensional structure of both peptides and can be functional. Due to the high expression of chloride and sodium channels in various cancer cells, MeICT/IMe-AGAP fusion peptide can be used as an effective agent to target both channels in cancers, simultaneously.

## INTRODUCTION

Scorpion venom contains a mixture of peptide toxins with diverse biological activities [1]. Scorpion toxins can be categorized into four types based on activity on ion channels: Na-channel toxins, K-channel toxins, Cl-channel toxins, and Ca-channel toxins [2]. These toxins also divided to long and short-chain toxins based on their sizes [3]. Although toxins are known for their deleterious effects on cells, some toxins have been detected with pharmaceutical activities against disease such as cancer [1]. Scorpion toxin peptides affect different pathways in cancer cells such as cell survival, cell death, angiogenesis, metastasis and invasion pathways [4]. 

Ion channels are involved in all molecular mechanisms of cancer cells. They regulate cell proliferation that is essential in cancer progression [5]. High expression and activity of sodium and chloride channels are associated with cancer invasion [6, 7]. Due to special interaction of scorpion toxins with ion channels, a lot of attention has been given to design drugs based on toxins and the list of scorpion-derived peptides that target cancer cells is expanding [4, 8]. 

In our previous studies, two toxins, MeICT and IMe-AGAP, were isolated from Iranian yellow scorpion, *Mesobuthus eupeus* [9, 10]. MeICT with 34 amino acids and four disulfide bonds contains three β sheets and one alpha helix (βαββ) in structure. MeICT is a Cl channel specific toxin that belong to short-chain toxins and composed of 35-38 amino acids with four disulfide bonds [9]. Our previous work showed MeICT inhibits cancer cell proliferation and downregulates the expression of genes involved in cancer progression [11]. This toxin can also interact with MMP2 that overexpressed in various cancer cells [12]. MeICT exhibited 81% similarity with CTX toxin isolated from *Leiurus quinquestriatus* scorpion which is a potent anti-tumor toxin used for diagnosis of glioma cells [13]. 

IMe-AGAP with 66 amino acids and four disulfide bonds also has βαββ scaffold structure. IMe-AGAP is a Na specific channel toxin belongs to long-chain scorpion toxins. Na-channel toxins consists of 60-76 amino acids and contains four disulfide bridges [10, 3]. IMe-AGAP shows strong anti-cancer activity on cancer cells (data are publishing). Bioinformatics studies represented that IMe-AGAP also binds to Na channels involved in pain and therefore it can be an analgesic peptide [14]. IMe-AGAP showed 93% similarity to AGAP toxin from *Buthus martensii* Karsch scorpion that inhibits proliferation of variety of cancer cells [15, 16]. Due to the anti-cancer efficiency of MeICT and IMe-AGAP, the aim of this study was design and construct a new chimeric peptide containing both MeICT and IMe-AGAP toxins to target different ion channels in cancer cells, simultaneously. This fusion peptide may target different cancer cells with strong inhibitory effect.

## MATERIALS AND METHODS

**Bioinformatics studies: **In the present study, protein Data Banks (http://www.ebi.ac.uk/ pdbe,https://zhanglab.ccmb.med.umich.edu/I-TASSER and http://www.sbg.bio.ic.ac.uk/phyre2) were used to obtain structure of proteins. Prediction of three-dimensional structures of MeICT, IMe-AGAP and fusion proteins MeICT/IMe-AGAP were analyzed by PEP-fold (http://biose rv. rpbs. univ- paris- diderot. fr/ servi ces/PEP- FOLD/). Fusion proteins with different linkers were assessed by chimera and Yasara workspace. Biochemical and physical properties of fusion peptide was studied by ProtParam web server (http://web.expasy.org/cgi-bin/protparam/ protparam). The allergenicity of peptides was determined by AllerTOP v.2.0 (https://www.ddg-pharmfac.net/AllerTOP)

**Linker and primer design:** Several different linkers were designed to connect two peptides MeICT and IMe-AGAP. Linkers were analyzed by Chimera and Yasara workspaces. Based on the selected linker sequence, two overlapping primers Flctagp (forward) and Rlmlct (revers) were designed by Gene Runner software. The sequences of primers were FLctagap: 5ˊGGTC CGTCGCCTGGTGTTCGTGATGGTTATATTGCCGA3ˊ and RLmlct: 5ˊCCGGGTGTTAC AGACAGACACAGCCCCAGGAGCGGACCAC3ˊ. The linker sequence was incorporated in these overlapping primers to connect MeICT and IMe-AGAP fragments.

**Amplification of MeICT and IMe-AGAP encoding fragments: **To amplify the MeICT gene, PCR reaction was performed by FscBH 5ˊGGATCCGGATCCGATGTGTATGCCTT GCTTTAC3ˊ and RLmlct primers on the plasmid containing MeICT gene according to the optimal condition: 94˚C for 5 min., followed by 35 cycles at 94˚C for 30˝, annealing at 55˚C for 45˝ and extension at 72˚C for 30˝. Similarly, IMe-AGAP sequence was amplified by RagpXH 5ˊCTCGAGGTCCATTTACGTTACCGCCAGAGCTCGTCAG3ˊ and FLctagp primers on the plasmid containing IMe-AGAP fragment with annealing temperature 58˚C. FscBH primer contained *Bam* HI enzyme cleavage site and RagpXH contained *Xho* I enzyme cleavage site that are defined as underline in sequences. After PCR reactions, 3 μl of products were run on 1% agarose gel to analyze amplified fragments.

**Construction and amplification of fusion peptide MeICT/IMe-AGAP using SOEing-PCR: **SOEing-PCR (splicing by over-hang-extension PCR) method was used to connect MeICT and IMe-AGAP encoding fragments. After amplification of MeICT and IMe-AGAP genes separately, the product of each component was diluted by 1/99. In SOEing-PCR reaction, MeICT and IMe-AGAP fragments were connected without primer in 15 cycles by condition: 94˚C denaturation for 45˝, 58˚C annealing for 45˝ and 72˚C extension for 45˝. Then FscBH and RagpXH primers were added to amplify the chimeric fragment MeI-CT/IMe-AGAP in the remaining 20 cycles at annealing temperature 56˚C. Pfu enzyme was used to reduce nucleotide errors in SOEing-PCR. Finally, the PCR product was applied to agarose gel.

**Cloning of MeICT/IMe-AGAP fragment in pET32Rh: **About 1 μg of MeICT/IMe-AGAP fusion fragment was digested by *Bam* HI and *Xho* I enzymes (Thermo-Fischer Scientific, USA) at 37˚C overnight. One micro gram of pET32bRh plasmid was also digested by both mentioned enzymes. Digested fragment and vector were purified from the gel. About 30 ng of purified MeICT/IMe-AGAP fragment and 20 ng of pET32bRh plasmid were ligated using T4 ligase enzyme (Takara, Japan) at room temperature for 2 hours. The ligation product was transformed into competent *Escherichia coli* TOP10 strain bacteria by heat shock method and grown in Luria-Bertani (LB)-agar medium containing ampicillin at 37˚C overnight. To obtain recombinant clones, colonies screened by colony-PCR method by T7 terminator and T7 promoter primers. For this purpose, several colonies were harvested with tips and dissolved in 10 µl of water separately, then boiled at 100˚C for 5 min and one µl of them was used as a template in PCR reaction with conditions of 94˚C for 5 min followed by 35 cycles, 94˚C for 1 min, 58˚C for 45˝ and 72˚C for 50˝. Plasmid was extracted from recombinant colony. To confirm the presence of desired sequence in the plasmid, PCR reaction was performed again on recombinant plasmid and 30 μl of product was sent to Gene Fanavaran Company for sequencing.

**Expression of recombinant MeICT/IMe-AGAP peptide: **To investigate the expression of chimeric protein, 10 ng of the recombinant plasmid was transformed into competent *E.coli* Bl21 bacteria by heat shock method. About 2 ml pre-culture were prepared from recombinant bacteria and added to 20 mL of LB medium containing ampicillin, then grown to OD_600_= 0.5. Expression of recombinant protein was induced by IPTG at concentration of 1 mM. The bacteria were grown at 28˚C for 4 and 6 h and then centrifuged at 4000 rpm, 4˚C for 10 min. The bacterial precipitate was lysed by 200 μl of TE solution (Tris–HCl 30mM, EDTA 1mM) and mixed well. Bacterial lysis was centrifuged at 15,000, 4˚C for 20 min. Both supernatant as soluble and the precipitate as insoluble phase were run in 12% SDS-PAGE gel.

## RESULTS

**Figure 1:**
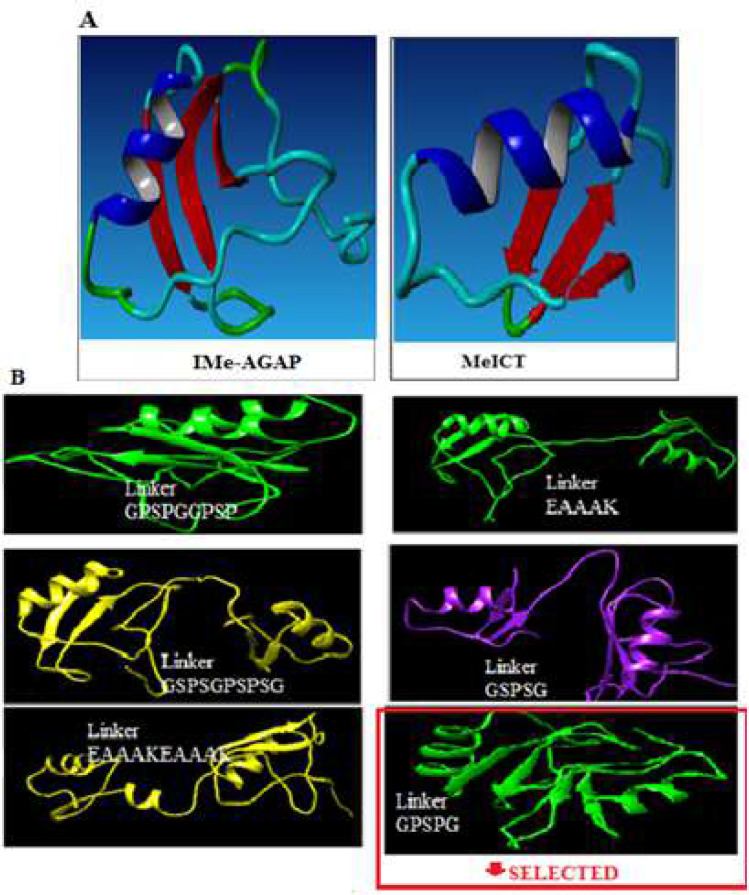
Three-dimensional structure of peptides. A) IMe-AGAP and MeICT structures. B) Three-dimensional structure of MeICT/IMe-AGAP peptides with different linkers by Chimera software. In selected part, the structures of both peptides are preserved and the distance between them is appropriate

In order to select the arrangement of MeICT and IMe-AGAP peptides in the chimeric fragment, the functions of different parts of the peptides were investigated. Due to the key function of MeICT amino-terminal and IMe-AGAP carboxyl-terminal, MeICT and IMe-AGAP were located in N-terminal and C-terminal, respectively and chimeric peptide was designed as MeICT/IMe-AGAP. To design fusion fragment, the three-dimensional structures of MeICT and IMe-AGAP peptides were obtained from Phyer2 and I-TASSER web servers. The three-dimensional structure of each peptide consists of an alpha helix and three antiparallel beta strands (Fig. 1A). In order to design a linker to connect MeICT and IMe-AGAP, rigid linkers were selected that prevent the interaction of two peptides with each other. With a rigid linker, each peptide keeps its structure and do not interfere the spatial space of other peptide. For this purpose, EAAAK, GSPSGPSPSG, GPSPGGPSPG, GPSPG, GSPSG, EAAAKEAAAK rigid linkers were investigated. The three-dimensional structures of the chimeric peptides with different linkers were obtained by Pep fold server and then analyzed by Chimera software. Figure 1B shows the final structure of the fusion peptides MeICT/IMe-AGAP with different linkers in Chimera software. Based on the obtained information, GPSPG was selected as the best linker. Both peptides had correct folding by this linker according to the Figure 1B. Therefore, the overlapping primers were designed to contain nucleotide sequences of this linker.

**Figure 2:**
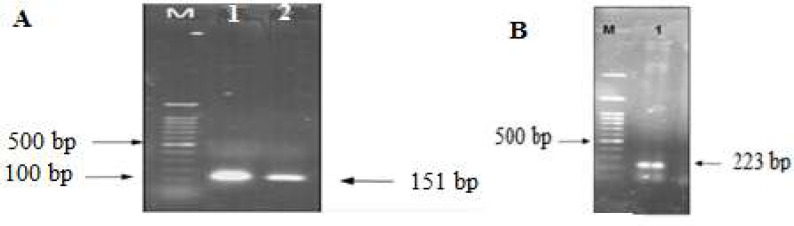
Electrophoresis of PCR products on 1% agarose gel. A) MeICT PCR product using FscBH and RLmlct primers, M: 100bp marker, 1 and 2: 150bp band. B) MeI-AGAP product using Flctagp and RagpXH primers M.100bp marker, 1 corresponds to 223bp MeI-AGAP sequence

MeICT and IMe-AGAP encoding fragments were amplified with tailing primers containing linker sequence which added linker sequences to the end of both genes. Figure 2 shows amplification of MeICT fragment with a size about 150bp and IMe-AGAP fragment with a size about 223bp. SOEing-PCR method was used to connect MeICT and IMe-AGAP genes. 

**Figure 3:**
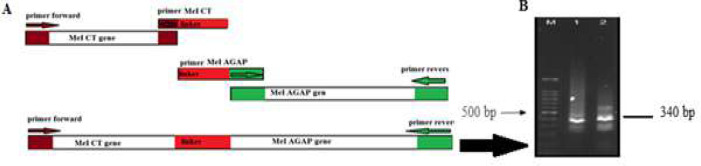
(A), Schematic diagram of MeICT and IMe-AGAP amplification and overlapping primers in SOEing-PCR. (B), Electrophoresis of fusion fragment MeICT/IMe-AGAP on 1% agarose gel. M: marker 100 bp 1 and 2: 340 base pair bands

Schematic figure 3A shows addition of linker sequence to each genes by primers and construction of chimeric fragment using PCR. After amplification of MeICT and IMe-AGAP fragments, SOEing-PCR was performed by FscBH and RagpXH primers to amplify chimeric fragment. PCR product around 340bp showed amplification of the MeICT/IMe-AGAP fusion fragment (Fig. 3B).

**Figure 4:**
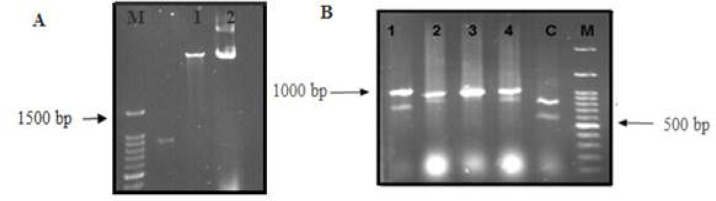
A) Plasmid extraction and digestion by *Xho*I and *Bam*HI enzymes. B) Electrophoresis of colony-PCR products by T7 Terminator and T7 promoter primers on 1% agarose gel in the presence of 100bp marker, 1-4: recombinant colonies with bands of 1000bp C: control containing the original plasmid with a band of 700bp

To clone the fusion fragment, plasmid pET32Rh was first extracted and digested with the restriction enzymes *Bam* H1 and *Xoh* I (Fig. 4A). Digested MeICT/IMe-AGAP fragment and vector were ligated using T4 ligase enzyme and were transformed into bacteria. For screening of colonies containing recombinant plasmid, a number of transformed colonies were analyzed by colony-PCR by T7-terminator and T7-promoter primers. Several recombinant colonies with a band around 1000bp were amplified that contained 340bp chimeric fragment and 650bp vector fragment (Fig. 4B).

**Figure 5:**
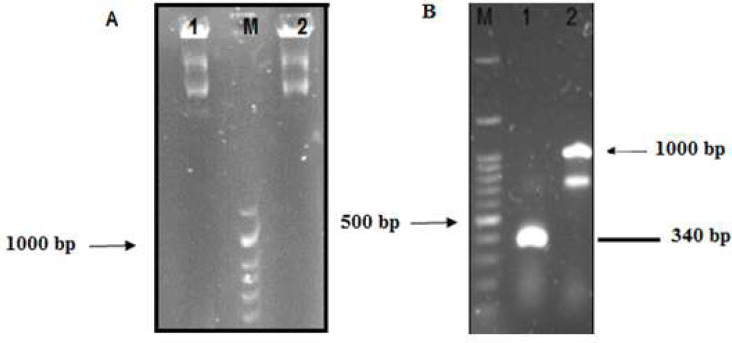
A) Recombinant plasmid extraction in lane 1 and 2, M: 100bp ladder. B) Amplification of the recombinant MeICT/IMe-AGAP fragment (lane 1) and 1000 bp fragment of vector by T7 primers

Bacterial culture was prepared from one recombinant clone and plasmid was extracted (Fig. 5A). PCR reaction was performed on the extracted plasmid by T7-ter, T7-pro and MeICT/IMe-AGAP primers. The results can be seen in part B of Figure 5.

**Figure 6:**
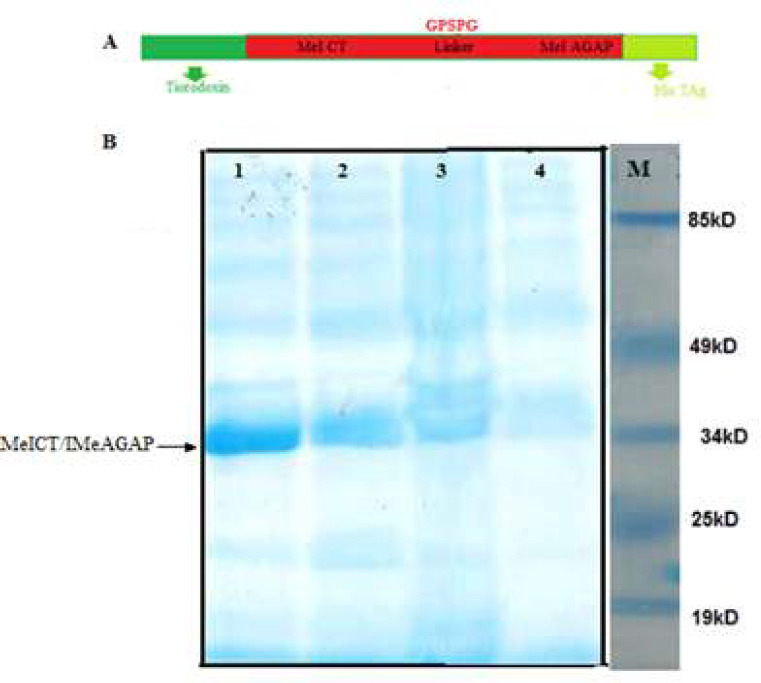
A) Schematic picture of expressed protein containing thioredoxin part, MeICT/IMe-AGAP and histidine tag. B, SDS-PAGE analysis of protein expression, lane 1: insoluble phase of protein extraction after 6 h induction, lane 2: insoluble phase of protein extraction after 4 h induction, lane 3: soluble phase of protein extraction after 6 h induction, lane 4: soluble phase of protein extraction after 4 h induction. 34 kD peptide is seen in all lanes. M: protein ladder

To investigate MeICT/IMe-AGAP expression, the recombinant vector was transformed into *E.coli* Bl21 bacteria and the protein expression was induced by IPTG at 28˚C. After four and six hour induction, the same amount of 10 milliliter culture was precipitate and lysed from both samples. To analyze in SDS-PAGE, 20 µl of each sample was used in gel. The MeICT/IMe-AGAP part contains 11.5 kDa (MeICT peptide about 4 kDa and IMe-AGAP peptide about 7.5 kDa). Thioredoxin fraction with 18.5 kDa weight is also added to N-terminal of recombinant protein in pET32Rh vector, therefore the recombinant protein with 30 kDa weight was detected in SDS-PAGE. The different parts of expressed protein containing histidine tag in C-terminal (Fig. 6A). The most of the recombinant protein were expressed in insoluble phases (Fig. 6B).

The chimeric peptide MeICT/IMeAGAP contains 105 amino acids. The weight of protein was estimated 11.47 kDa. This protein includes 10 negative and 13 positive charge residues, therefore its net charge is positive. The protein instability index was determined 36.45 by ExPASY web server. Since the instability index below 40 indicates the stability of the protein, MeICT/IMeAGAP fusion peptide is a stable protein. The grand average hydrophobicity was estimated -0.58. Mean positive hydrophobicity indicates hydrophobic protein and negative hydrophobicity indicates hydrophilic protein, thus, our peptide is hydrophilic. The isoelectric point of the chimeric protein is in the neutral range of 8.12 that is the pH at which the protein charge is zero. The degree of solubility of this protein is 0.53 of 1. The half-life of the chimeric peptide is more than 10 hours in *Escherichia coli* host cells, more than 20 hours in *Saccharomyces cerevisiae* yeast cells, and 30 hours in vitro based on ProtParam web server. The comparison of three peptide MeICT/IMeAGAP, MeICT and IMeAGAP properties has been shown in Table 1. All three peptides may be allergen based on estimation of AllerTOP server.

**Table 1:** Characterization of peptids

	**MeICT/IMeAGAP**	**MeICT**	**IMeAGAP**
**Number of amino acids**	105	34	66
**Molecular weight**	11469.09	3767.49	7324.21
**Theoretical pI**	8.12	8.16	7.64
**Negatively charged residues**	10	2	8
**Positively charged residues**	13	4	9
**Estimated half-life**	30 hours (in vitro). >20 hours (yeast, in vivo). >10 hours (Escherichia coli, in vivo)	100 hours (in vitro). >20 hours (yeast, in vivo). >10 hours (Escherichia coli)	100 hours (in vitro). >20 hours (yeast, in vivo). >10 hours (Escherichia coli, in vivo)
**Instability index**	36.45 stable	45.84 unstable	25.18 stable
**Grand average of ** **hydropathicity**	-0.586	-0.221	-0.745

## DISCUSSION

**Figure 7:**
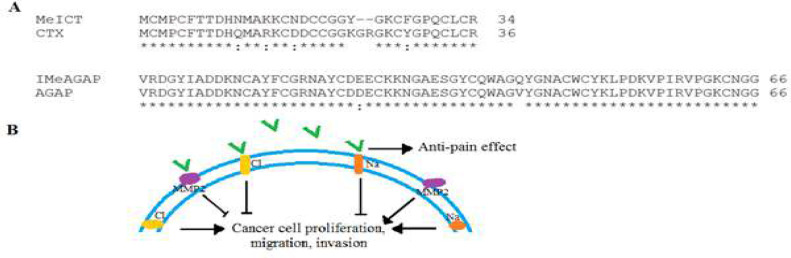
A) Alignment of MeICT with CTX and IMeAGAP and AGAP peptides. The similar amino acids have been determined by star. B) Possible action of chimeric peptide to inhibition of both Na and Cl channels on cell membrane. Peptide has been shown as green lines

Ion channels play key roles in cancer progression and affect various stages of cancer including proliferation, survival, migration, metastasis and angiogenesis [5, 17]. Hence, targeting ion channels has been considered as an effective method in cancer therapy. Scorpion toxins can specifically target different ion channels in cancer cells [8, 18]. The expression of chloride channels increases in various cancers such as glioma [6]. High expression of sodium channels has been also observed in the proliferation and migration of various cancer cells such as colon, breast, and lung cancers [19]. Both Cl and Na channels are appropriate therapeutic goals for tumor targeting. In our previous studies, two anti-cancer scorpion toxins were isolated that affect different pathways in cancer. MeICT and IMe-AGAP toxins target chloride and sodium channels, respectively [9-11]. Therefore, these peptides can target various cancers, depending on the type of channel involved. Among scorpion toxins, MeICT and IMe-AGAP show high similarity to chlorotoxin (CTX) and AGAP that are two strong anti-cancer toxins (Fig. 7A). CTX and AGAP are most known toxins that target chloride and sodium channels in cancer cells, respectively [13, 16]. They also affect other cancer pathways and effectively inhibit cancer progression [15]. 

Although peptide toxin are potent agents for targeting cancer, due to low molecular mass, they are prone to fast renal elimination. One suitable approach to prolonging plasma half-life of small toxins is to increase the effective molecular mass via coupling them to longer proteins [20]. Larger size of the chimeric peptide MeICT/IMe-AGAP increase its plasma half-life compare to MeICT and IMe-AGAP. So far, there was not any studies about an agent targeting two ion channels simultaneously. In this study for the first time, we tried to produce a chimeric peptide from two toxins with different function that can target a wide range of cancers. In spite of Cl channels, MeICT also interact to MMP-2 that is overexpressed in various metastatic cancers [21, 22]. In addition, IMe-AGAP like AGAP can represent analgesic activity [14]. Therefore, this chimeric peptide as a cancer inhibitor may also relief pain in cancer patients. The schematic Figure 7B indicates the probable functions of chimeric peptide MeICT/IMe-AGAP.

In order to construct a fusion protein of IMe-AGAP and MeICT, several rigid linkers were considered to find a stable folding of both peptides. Examination of linkers by bioinformatics methods led to the selection of GPSPG linker for chimeric peptides [23, 24]. This linker contains five residues including two glycine, a small, neutral amino acid that has no effect on protein structure. Hence, two residues of glycine at both sides of linker do not impress structure of fusion protein and do not create a spatial barrier. Serine as a polar amino acid was used for peptide flexibility [24]. Two prolines were used to create rigid form of the linker and to make a suitable distance between two fused peptides. This linker with small size does not cause spatial barrier or structural change on peptides [23, 24]. Using this linker, two peptides were spaced well apart and they maintain their functional and spatial structure. Bioinformatics studies confirmed the preservation of MeICT and IMe-AGAP structures by mentioned linker. 

SOEing-PCR method was used to join MeICT and IMe-AGAP gene sequences in two steps. For this purpose, overlapping primers were designed containing linker sequence. At first, two encoding fragments were amplified with overlapping primers. In second stage PCR, two amplified fragments were mixed and join without primers to construct MeICT/IMe-AGAP fragment, then amplified by adding specific primers. MeICT was embedded in the N-terminal and IMe-AGAP located in the C-terminal of fusion peptide, due to the function of the carboxyl terminus of Na channel toxins and the amino terminus of the MeICT [25, 26]. This design resulted to availability of active parts of fusion peptide that inhibit their overlap. 

Due to the high number of disulfide bonds in MeICT/IMe-AGAP fusion peptide, four disulfide bond for each peptide, pET32Rh vector was used for cloning. This vector contains thioredoxin, which binds to N-terminal of recombinant peptide. Thioredoxin part catalyzes the correct formation of disulfide bridges in the cytoplasm and increases the solubility of recombinant proteins. Binding to the protein partners such as thioredoxin decrease the toxicity of the recombinant peptides and increases the stability of small peptides by reducing proteolysis [27, 28]. However, the number of 8 disulfide bonds in MeICT/IMe-AGAP decreased the soluble expression of this protein and most of the peptide was detected in insoluble part. According to SDS-PAGE results, it seems that MeICT/IMe-AGAP can be produced in large scale, however the future studies to increase the soluble expression of fusion peptide can be performed by changing the culture conditions or using denaturing agents. The purified chimeric peptide can be used via injection to target cancer cells that overexpress both Na and Cl channels. MeICT/IMe-AGAP fusion peptide could be a potential target for cancer cells, which future studies on this fusion peptide will determine how it works.

### Acknowledgements:

We thanks Shahrekord University for supporting this work.

### Conflict of Interest:

We declare that we have no conflict of interest.
